# Putting the waste out: a proposed mechanism for transmission of the mycoparasite *Escovopsis* between leafcutter ant colonies

**DOI:** 10.1098/rsos.161013

**Published:** 2017-05-17

**Authors:** Juliana O. Augustin, Talitta G. Simões, Jan Dijksterhuis, Simon L. Elliot, Harry C. Evans

**Affiliations:** 1 Department of Entomology, Universidade Federal de Viçosa, CEP 36.570-900, Viçosa, MG, Brazil; 2 CBS-KNAW Fungal Biodiversity Centre, PO Box 85167.3508 AD, Utrecht, The Netherlands; 3 CAB International, E-UK Centre, Egham, Surrey TW20 9TY, UK

**Keywords:** attine ants, *Escovopsis*, horizontal transmission, *Leucoagaricus*, phoresy, spore dormancy

## Abstract

The attine ant system is a remarkable example of symbiosis. An antagonistic partner within this system is the fungal parasite *Escovopsis*, a genus specific to the fungal gardens of the Attini. *Escovopsis* parasitizes the *Leucoagaricus* symbiont that leaf-cutting ants (*Acromyrmex*, *Atta*) have been farming over the past 8–12 Myr. However, it has been a puzzle how *Escovopsis* reaches its host. During a seasonal survey of nests of *Acromyrmex subterraneus subterraneus* in Atlantic rainforest in Brazil, *Escovopsis* was detected in all the sampled fungal garden waste tips or middens (*n* = 111). Middens were built strategically; always below the nest entrances. Here, we report the first evidence of a putative mechanism for horizontal transmission of *Escovopsis* between attine colonies. It is posited that leaf-cutting ants pick up the spores from soil and litter during foraging and vector the mycoparasite between attine colonies. Field and laboratory experiments, using *At. laevigata* and *Ac. subterraneus subterraneus*, confirm that *Escovopsis* spores are phoretic, and have an inbuilt dormancy, broken by the presence of their *Leucoagaricus* host. However, in the coevolutionary arms race, *Atta* ants may lose out—despite most species in the genus investing in a more advanced waste disposal system—due to the insanitary habits of their *Acromyrmex* neighbours.

## 1. Introduction

Fungus-growing ant symbiosis has emerged as a model system for the study of parasitism in complex insect societies. In this system, leaf-cutting ants tend their domesticated fungus *Leucoagaricus* (Basidiomycota, Agaricales) [1], providing it with optimum conditions for growth. Over evolutionary time, and this has been estimated at 8–12 Myr [1], the mutualistic fungal garden has become the main food source of the ant colonies, on which the larvae and queen feed exclusively [2]. This obligate mutualism is exploited by a fungal parasite of the genus *Escovopsis* (Ascomycota: Hypocreales): one of the non-mutualistic fungi most frequently isolated from and exclusive to attine nests, and which has been implicated in colony debilitation; thus, posing a direct threat to ant fitness [3,4]. However, despite a steady flow of publications on the biology of *Escovopsis* over the past 10–15 years [5–12], there is still no evidence of how this mycoparasite reaches its fungal host within the ant nest, which limits our understanding of the ecological and evolutionary dynamics of the attine ant–microbe symbiosis.

Sampling of newly founded *Atta* colonies has not revealed, thus far, the presence of the mycoparasite [3,13,14] and, although vertical transmission allied to initial dormancy cannot be ruled out, these data strongly favour horizontal rather than vertical transmission of *Escovopsis*. As previous studies suggest [4,10,11], a possible mechanism is that other arthropods (inquilines) or foraging leafcutters accidentally pick up fungal spores and carry them into the nest. However, this scenario would require the mycoparasite to sporulate outside of the ant colony. Here, we consider the key question of how the fungus effects transmission between ant colonies.

During field surveys in southeast Brazil, we found *Escovopsis* sporulating on an external fungal garden waste tip—hereafter, designated as a midden—of the leaf-cutting ant *Acromyrmex subterraneus subterraneus*. To determine if this was an isolated event, or represented something more frequent and significant within the fungal life cycle, we investigated midden phenology in colonies of *Ac. subterraneus subterraneus* within a forest ecosystem, and complemented this with laboratory and field experiments to elucidate the potential transmission mechanism of the *Escovopsis* mycoparasites encountered.

## 2. Material and methods

### 2.1. Phenology and sampling of middens

**Figure 1.**
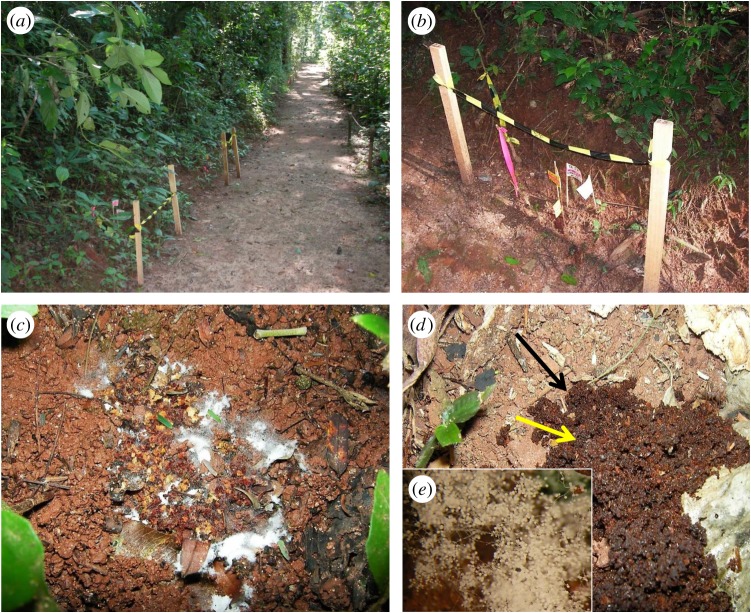
Aspects of the study site, sampling protocol and middens. View of study area along a walking trail in Atlantic rainforest (Minas Gerais, Brazil), with nest sites of *Acromyrmex subterraneus subterraneus* marked by posts (*a*), and (*b*) the fungal garden waste tips or middens—always below the nest entrance on sloping ground—flagged and labelled sequentially, (*c*) mass sporulation (blooming) of *Escovopsis* on midden, (*d*) midden with no macroscopic evidence of *Escovopsis*—note that the midden (black arrow) stands out from the background soil—but microscopic examination reveals colonization by and early sporulation of *Escovopsis* (inset, *e*).

We identified and marked 34 nests of *Ac. subterraneus subterraneus* along a well-maintained walking trail (approx. 250 m in length, figure 1*a*) in Atlantic forest (Mata Atlântica): a seasonal, subtropical, semi-deciduous, montane forest, forming part of a small reserve (Mata do Paraíso) near Viçosa in the southeastern region of the Zona da Mata Mineira, State of Minas Gerais, Brazil (20°48′07^″^ S, 42°51′31^″^ W, 680 m a.s.l.). Colonies were inspected weekly from late February to late June 2011 and middens were mapped over time. Because individual colonies produce multiple middens, these were marked sequentially (figure 1*b*), in order to record the number of middens produced by each colony. This allowed us to monitor the history of each midden, including mass sporulation events, when fungal overgrowth was clearly visible (blooming). All middens—with or without blooming (figure 1*c,d*)—were marked and provisional identification of *Escovopsis*, with its distinctive sporogenesis [12], was made using a hand lens (×20). Samples (approx. 1 ml) of all middens were collected in sterile plastic tubes for laboratory examination. Distances between nests were taken with the aid of a measuring tape, as well as the distances between middens and nest entrances. Weekly rainfall data were obtained from the forest reserve meteorological station, approximately 0.5 km from the study site. In addition, ad hoc observations of midden building by *Ac. subterraneus subterraneus* were made along the same trail from October 2012 until May 2013.

### 2.2. Laboratory examination of midden samples

Midden samples were decanted into sterile plastic Petri plates (5 cm diameter) and examined using a stereomicroscope (Olympus SZX10). Sporulating colonies were transferred with the aid of a sterile hypodermic needle to glass slides containing a drop of 1% acid fuchsin and examined using a light microscope (Olympus RX51) to confirm the presence of *Escovopsis*. Midden samples with no evident fungal colonization were treated similarly but subjected to a more intensive screening. After confirmation of *Escovopsis*, we characterized the former samples as blooming—visible with the naked eye (figure 1*c*)—and the latter samples as cryptic—visible only under the stereomicroscope (figure 1*d*).

### 2.3. Fungal isolation from middens and identification

With the aid of a stereoscopic microscope, fragments of sporulating mycelium were removed with a sterile hypodermic needle and streaked directly onto Petri plates containing either tap water agar (TWA) or potato carrot agar (PCA), supplemented with penicillin and streptomycin sulfate. Plates were examined daily and colonies were subcultured on PCA or potato dextrose agar (PDA), as they appeared. Sporulating structures from mature colonies were mounted in 1% acid fuchsin and examined using an Olympus light microscope (BX 51), fitted with a MicroPublisher 33RTVQ and the images were captured with an Olympus E 330 camera.

### 2.4. Spore morphology using scanning electron microscopy

To facilitate a better interpretation of spore morphology, particularly wall ornamentation, scanning electron microscopy (SEM) was employed. Material was selected from cultures under a binocular microscope, excised with a surgical blade as small agar blocks (3 × 3 mm), and transferred to a copper cup for snap-freezing in nitrogen slush. Agar blocks were glued to the copper surface with frozen tissue medium (KP-Cryoblock, Klinipath, Duiven, The Netherlands) mixed with one part colloidal graphite (Agar Scientific, Stansted, UK). Samples were examined in a JEOL 5600LV scanning electron microscope (JEOL, Tokyo, Japan), equipped with an Oxford CT1500 Cryostation for cryo-electron microscopy (cryoSEM).

### 2.5. Spore dormancy *in vitro* bioassay

To investigate spore dormancy in *Escovopsis*—a phenomenon encountered regularly during initial isolation from middens, as well as during subculturing from ageing colonies—and the potential interactions with its host in the fungal garden, an *in vitro* bioassay was undertaken. Mature spores from old colonies (more than three months, left to dry out at 25°C in an incubator so that vegetative growth would be killed and spores could easily be harvested) of type strains of three *Escovopsis* species (*E. weberi*, *E. moelleri* and *E. lentecrescens*; note that all three types were isolated originally from the municipality of Viçosa, the latter two identified in a recent study from our group [12]) were harvested and stored separately in sterile containers for use as inoculum. Petri plates (5 cm diameter) containing TWA, PDA or PCA were inoculated centrally with mycelial discs (4 mm diameter) from young colonies of *Leucoagaricus* (originally isolated from a colony of *Atta sexdens rubropilosa* from Viçosa) grown on PDA. *Escovopsis* spores were suspended in sterile distilled water and applied to the plates in droplets (inocula) with a hypodermic syringe (100 µl per droplet; 1 × 10^6^ spores ml^−1^), at four equidistant points, 1.5 cm from the central disc. Five replicates were used per agar medium (=15 plates/60 inocula per species of *Escovopsis*). The experiment included two controls, in which the central *Leucoagaricus* discs were not present, but were rather substituted by discs of sterile PDA (control 1); or, discs taken from PDA cultures of an agaricaceous fungus, *Moniliophthora perniciosa*, that is related to *Leucoagaricus* and was isolated here from witches' broom disease of cacao [15] (control 2). The experiment was examined weekly for evidence of growth and was terminated after 30 days. Differences in germination between controls without *Leucoagaricus* and plates with this fungus were analysed using a *χ*^2^-test. At day 30, one-third of the ungerminated control plates (for all three *Escovopsis* species and culture media) were seeded with mycelial discs from a young colony of *Leucoagaricu*s, one-third were seeded with plugs from an older colony of *Leucoagaricus*, while the remainder were left without the ant mutualist. The aim was to see if this new stimulus would now trigger germination and growth.

### 2.6. Proof of phoresy

Initial *in vitro* laboratory experiments, in which ants of the genera *Atta* and *Acromyrmex* were placed in Petri plates containing either cultures of *E. moelleri* or sterile sand impregnated with *E. moelleri* spores, showed that the spores attached readily to the bodies and legs of the ants, often impeding their subsequent movements. This was considered to be an artificial and potentially misleading method of proving phoresy and it was decided that proof of concept would be better tested under more natural conditions. Note that this *Escovopsis* species was used as its spores are significantly larger than other *Escovopsis* species and have a prominent cap, greatly facilitating their detection and identification on the ant appendages.

A laboratory study was conducted with three lab-reared colonies of *Ac. subterraneus subterraneus* (originally from the municipality of Viçosa) and two isolates of *E. moelleri* (both also from Viçosa; one the type strain [12] and one that has not been deposited). Sterile filter paper discs (1.5 cm diameter), that had been immersed in spore suspensions (2.5 × 10^6^ and 2.5 × 10^7^ conidia ml^−1^ of sterile distilled water), prepared from old colonies (more than three months) of *E. moelleri*, were placed in Petri dishes (15 cm diameter) and ants were placed in these dishes for 5 min). These were then collected with fine forceps (without touching the legs, and with forceps swabbed with cotton wool impregnated with 70% ethanol between each collection) and stored as described below. Further foraging ants were collected from the colony to serve as a control.

**Figure 2.**
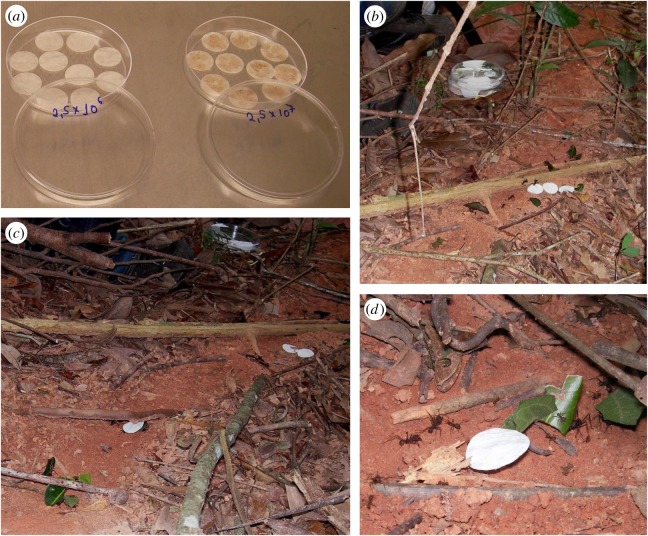
Phoresy field experiment. Experimental set-up showing discs of filter paper impregnated with spore suspensions of *Escovopsis moelleri* drying in a laminar flow (*a*), discs *in situ* on an ant trail of *Atta laevigata* (*b*), and discs being removed and carried towards nest entrance (*c,d*).

For an equivalent field study, nests of the leaf-cutting ant *At. laevigata*—chosen because of its daytime rather than nocturnal activity, and its construction of prominent trails—were located in a small private reserve of Atlantic forest, ‘Mata do Seu Nico’, 20°45′23^″^ S and 42°52′23^″^ W, 750 m altitude, near Viçosa. The experiment was carried out in July 2015 during the dry season when midden building is minimal and thus contamination of the trails by spores of *Escovopsis* would be unlikely. Discs immersed in spore suspensions (as above) were placed three abreast on the ant trails (figure 2*b*). Any ants which were observed to come into contact with the discs were monitored and collected using fine forceps at a site approximately 4 m further down the trail. Ants were collected from the trails prior to the experiment to serve as controls.

In both assays, ants were placed individually in sterile tubes, transferred to the laboratory and stored in a deep freezer to await examination. The lower leg section (tarsus) of the ants was dissected, mounted in 1% acid fuchsin and examined using an Olympus light microscope (BX 51), fitted with a MicroPublisher 33RTVQ and the images were captured with an Olympus E 330 camera.

## 3. Results

### 3.1. Phenology and sampling of middens

**Figure 3.**
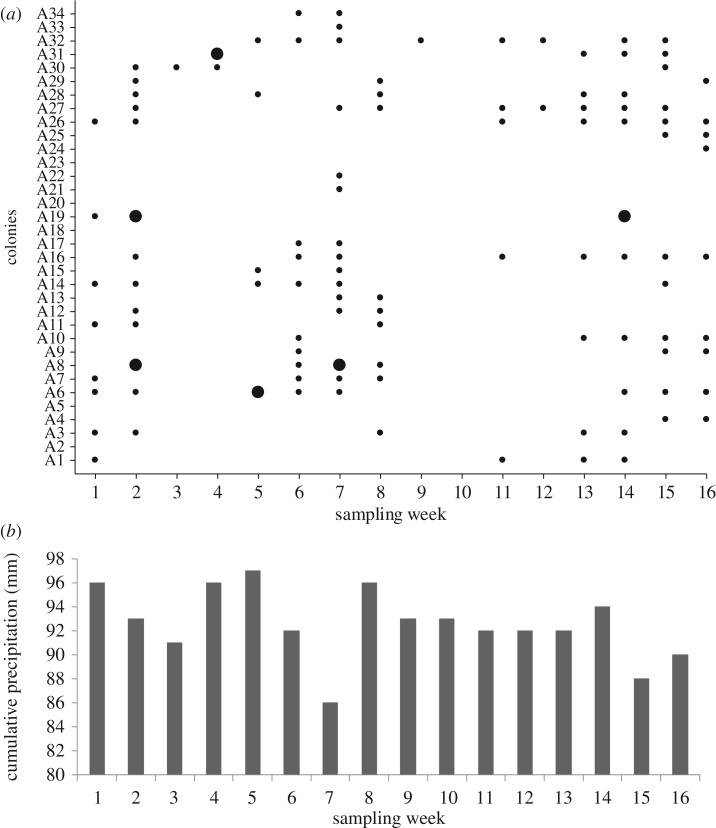
Phenology of middens. (*a*) Frequency of middens (*n* = 111) produced by 34 field colonies of *Acromyrmex subterraneus subterraneus* over a 16-week period from February to June 2011—small dots represent midden samples in which *Escovopsis* was confirmed following microscopic examination (= cryptic *Escovopsis*), large dots represent midden samples from which we observed *Escovopsis* sporulating macroscopically in the field (=blooming *Escovopsis*), (*b*) cumulative weekly rainfall data from the sampling site during the midden-sampling period.

**Figure 4.**
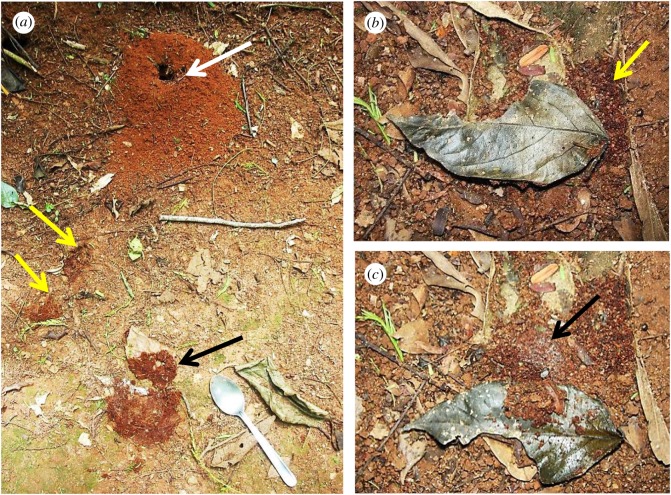
Location of middens. (*a*) Funnel-shaped nest entrance of *Acromyrmex subterraneus subterraneus* (white arrow) with two small middens on slope below (yellow arrows), and a larger midden further down the slope, previously hidden beneath a fallen leaf that has been turned over (black arrow); note the typical sparse leaf coverage of the nest site, (*b*) another nest site with fallen leaf and midden-building at edge (yellow arrow), (*c*) same leaf, overturned to reveal the main midden hidden beneath and the central area overgrown by *Escovopsis* (black arrow).

Most colonies of *Ac. subterraneus subterraneus* produced middens continuously over the sampling period (more than 85%, *n* = 34). Significantly, *Escovopsis* was found in every midden sample (*n* = 111; figure 3*a*), and these were classified as either blooming (5.4%, *n* = 6) or cryptic (94.6%, *n* = 105). The rarer blooming was always the faster-growing *weberi* or cylindrical type; the much commoner cryptic instances were of the slower-growing *aspergilloides* (=*lentecrescens*) or globose type. The nest entrance mounds were small (109.46 ± 8.79 cm^3^; figure 4*a*), as well as the middens themselves (3.04 ± 1.17 cm^3^). This can be an indication that the ant colonies were young [16]. However, the frequent rain over the study period eroded the nest entrances, as well as the middens, which were continually being rebuilt; indicating that the colonies were healthy. Because the ant colonies were located along the sloping gradient of an open trail (figure 1*a,b*)—with the nest entrances towards the top of the slope and the middens always below (figures 3 and 4)—the middens could readily be located, especially as their reddish-brown colour contrasted sharply with the soil. Colonies were separated from each other on average by 12.55 m (s.e. = 8.43). Each colony produced between 1 and 8 middens which were built, on average, 9.02 cm (s.e. = 2.36) below the nest entrances. The mean number of middens constructed around or under the sparse leaf litter coverage was 18.6 (s.e. = 6) (figure 4), compared with 48 (s.e. = 16) for those deposited in the open. Blooming *Escovopsis* on middens occurred sporadically, with too low a frequency to discern any relationship with the rainfall data (figure 3*a,b*). Moreover, sporulation was not only rapid—occurring within 4 days of fungal garden waste being deposited by workers—but also ephemeral, as middens were short-lived (less than 7 days) due to the frequent rainfall: week 9, for example, shows only one midden found in the sampling area, followed by none in week 10. Colonies started rebuilding middens from week 11 onwards following a period of rain (figure 3*a*). However, it is difficult to draw any firm conclusions between midden building and rainfall patterns over the relatively short time frame of the survey.

**Figure 5.**
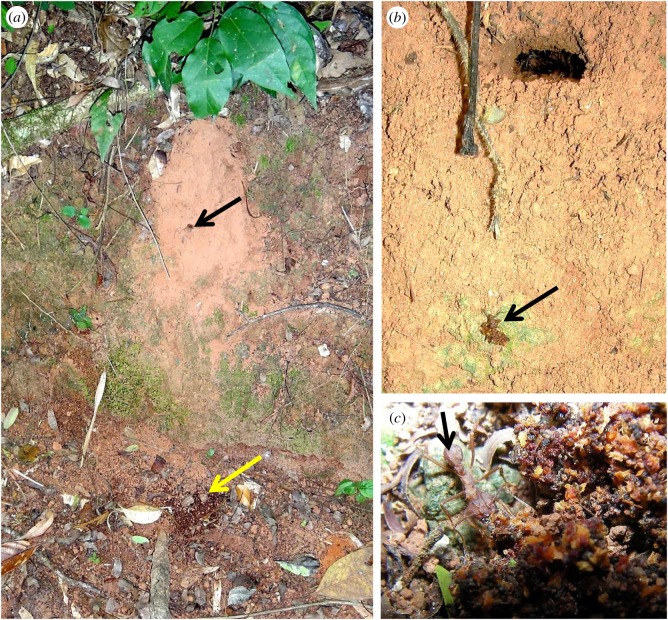
Construction of middens. (*a*) Nest site of *Acromyrmex subterraneus subterraneus* on an almost vertical slope showing an entrance (black arrow) with a midden below (yellow arrow), (*b*) *Acromyrmex* worker (black arrow) transporting garden waste from the nest to the midden below, (*c*) same worker (black arrow) incorporating the garden waste into the midden.

Supplementary observations and sampling, undertaken in the study area from October 2012 until May 2013, supported the data obtained during 2011: the middens were always constructed below the nest entrance and all were colonized—cryptically or, less commonly, overgrown—by *Escovopsis*. Furthermore, monitoring of the activities of the midden workers revealed that the ants transported the garden waste from the nest entrance all the way to the chosen disposal site. Even when there often appeared to be an easier option to drop the waste from above, especially on steeply sloping nest sites (figure 5*a*), attendant ants carried the waste to the midden (figure 5*b*) and carefully worked it into the midden (figure 5*c*).

### 3.2. Laboratory examination of midden samples

**Figure 6.**
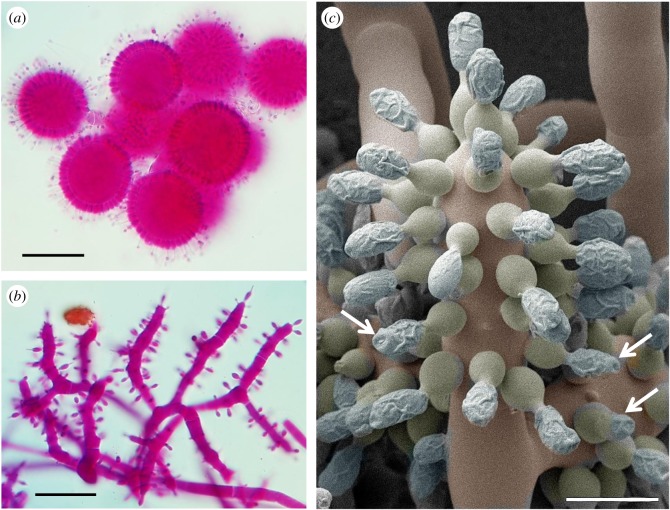
Morphology of *Escovopsis*. Typical sporulating structures of *Escovopsis* taken directly from a cryptic midden sample (figure 1*d*), showing globose vesicles of (*a*) *E. aspergilloides*-type and cylindrical vesicles of (*b*) *E. weberi*-type, (*c*) CryoSEM of *E. moelleri* showing the large conidia produced on flask-shaped phialides from a clavate-cylindrical vesicle and developing distinctive wall ornamentation, apparently mucilaginous, with apical caps (arrows). Scale bars, (*a,b*) 30 µm, (*c*) 12 µm.

**Figure 7.**
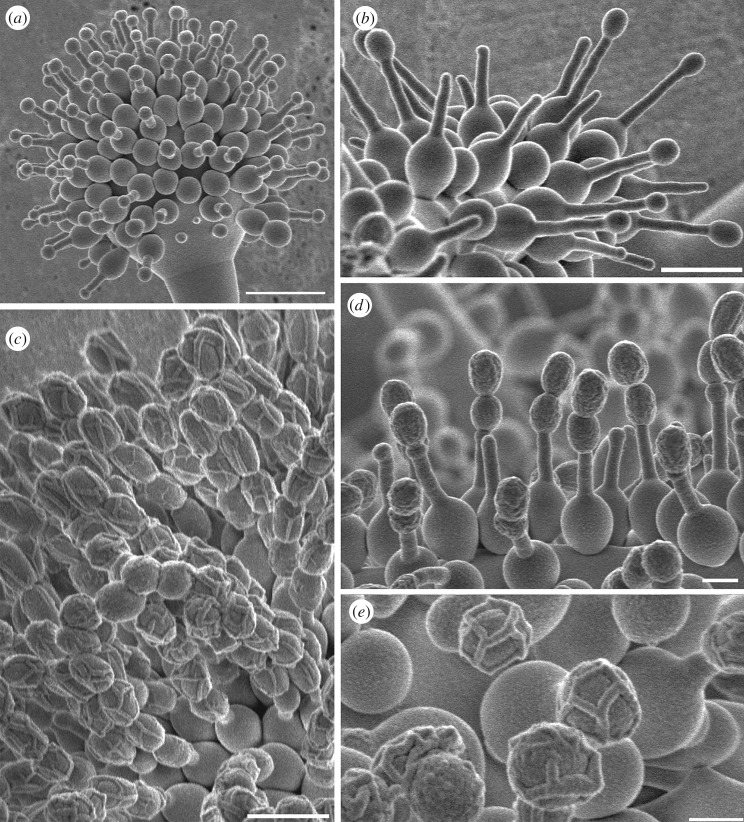
SEM study of *Escovopsis* species. To illustrate the development of and variation within spore ornamentation, in (*a*) *E. aspergilloides* the young spore stage on the phialides lacks ornamentation and (*b*) mature spores in chains with lattice-like ornamentation, (*c*) *E. weberi* early spore stage and (*d*) later stage with coating or ornamentation covering the surface, (*e*) mature spores of *Escovopsis* sp. showing a grid-like ornamentation. Scale bars, (*a*) 10 µm, (*b,c*) 5 µm, (*d,e*) 2 µm.

The *Escovopsis* morphotypes that sporulated on the middens fell into either the *aspergilloides*-type (globose vesicles; figure 6*a*), or the *weberi*-type (clavate-cylindrical vesicles; figure 6*b*) [12], with the former being predominant. Light microscopy revealed that the maturing spores become thick-walled and pigmented with a distinctive ornamentation on the lateral walls and often the development of an apical cap. The latter feature is especially evident in the larger-spored forms, not found in this study but described previously, notably *Escovopsis moelleri* [12], as shown in figure 6*c*. Based on cryoSEM images, the ornamentation—in the form of filaments forming lattice- or grid-like patterns on the spore surface (figure 7)—is considered to be mucilaginous in nature. This may explain the reason for the mature spores to clump together rather than to disperse separately as in dry-walled spores. Attempts to obtain cultures from old (brown) colonies of *Escovopsis*—characterized by the presence of collapsed vesicles and heavily pigmented spores in midden samples—often proved to be difficult. *Escovopsis* isolation was most successful when younger, non-pigmented (white) fungal colonies were selected instead. This situation offered further evidence of a dormancy mechanism in operation.

### 3.3. Spore dormancy *in vitro* bioassay

**Figure 8.**
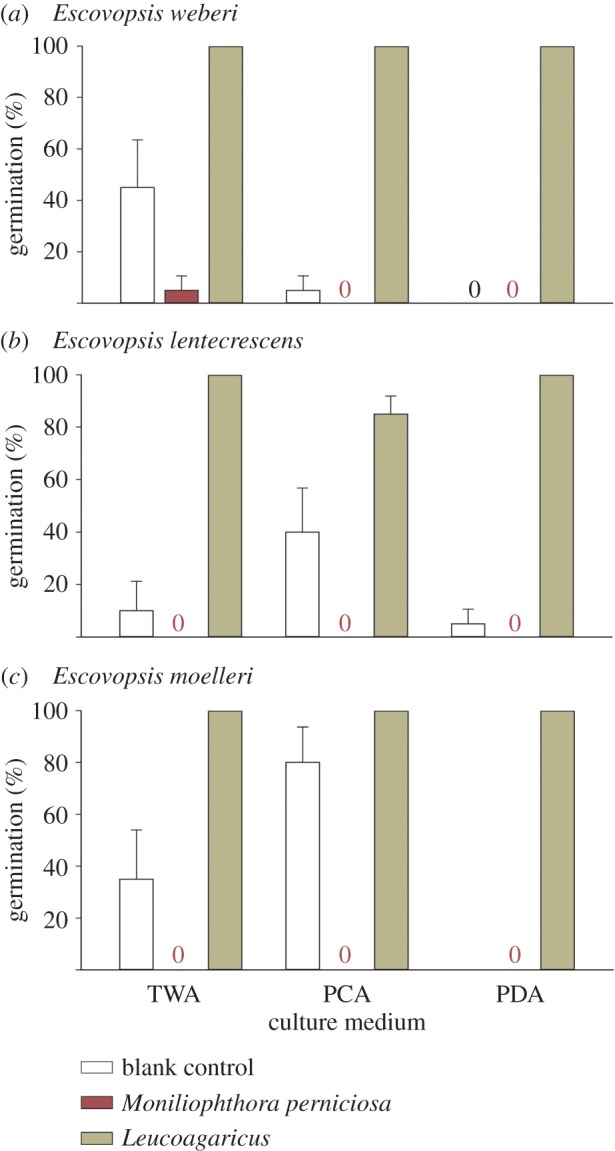
*Escovopsis* germination assay. Germination of spores of three species of *Escovopsis* on plates with three culture media and in the presence of discs of potato dextrose agar with a blank control (no fungus, only agar), the basidiomycete *Monoliniophthora perniciosa* on agar or the host of *Escovopsis*, *Leucoagaricus*, on agar. Germination percentages are by records of growth following germination on Petri dish quadrants, such that recorded values were 0, 25, 50, 75 or 100%. Presented are means of these values from five such plates for each experimental situation, while error bars are standard errors of these means.

**Figure 9.**
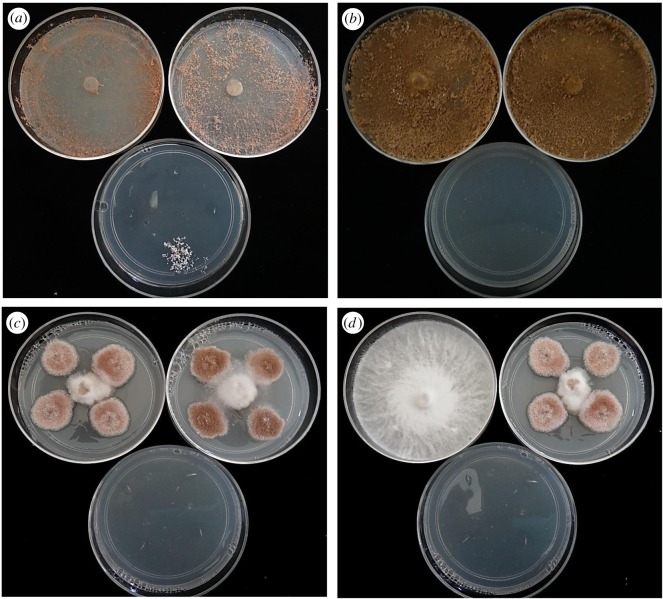
Results of spore dormancy assay. Showing (*a*) *Escovopsis weberi* covering the plates (upper) after growth from all the inoculum points surrounding the central inoculum plug of the *Leucoagaricus* fungal symbiont, and control 1 (lacking the symbiont, lower plate), with three non-germinating inocula and one inoculum point initiating growth but significantly slower than in the presence of *Leucoagaricus*, (*b*) heavily pigmented colonies of *E. moelleri* growing from all the inoculum points and covering the fungal symbiont (upper plates), but no growth from any of the four inoculum points in the control (lower plate), (*c*) *E. lentecrescens* growing slowly from all the four inocula surrounding the central *Leucoagaricus* inoculum plug (upper plates) but no growth in the control (lower plate), (*d*) the agaric *Moniliophthora perniciosa* completely overgrowing the plate from the central inoculum plug (control 2), with no evidence of germination or growth of *Escovopsis* from the four inoculum points (left plate); in sharp contrast with the *E. lentecrescens* reaction in the presence of *Leucoagaricus* (right plate), and no growth from any of the control inocula (lower plate).

The mycoparasite *Escovopsis* germinated within 14 days and grew in almost all (177 of 180) of the experimental situations in which the *Leucoagaricus* symbiont was present on the same agar plate (figure 8). This was in sharp contrast to control 1 (lacking the symbiont) in which only 20/45 plates and 44/180 inocula showed growth of *Escovopsis* (figures 8 and 9*a–c*) ($\chi_{1}^{2}=207.3,$ *p* < 0.01). While in the presence of the related agaric *Moniliophthora perniciosa* (control 2, figure 9*d* and figure 8), both germination and establishment of *Escovopsis* were inhibited and growth was restricted to a single plate: all the spore inocula were overgrown by the white mycelial mat or subiculum of *M. perniciosa*. Although we did not measure growth rates, there was clear evidence during the observation period that *Escovopsis* germination was quicker and growth was considerably faster in the presence of its host than in the control without *Leucoagaricus* (figure 9*a*). Moreover, in order to test further the robustness of the results, mycelial discs of *Leucoagaricus* were placed in the centre of two-thirds of those plates in control 1 where *Escovopsis* (all three species) had failed to germinate after termination of the experiment. The mycoparasite emerged within 7–14 days on the plates that had been seeded with the old colony of *Leucoagaricus*, and between 15 and 30 days on plates seeded with the young colony (with no notable variation between species or culture media). Growth occurred from all the spore inocula (60/60). However, no growth was detected in the control treatment lacking *Leucoagaricus* (eight plates, 0/32 inocula, figure 8), after an extended two-month observation period.

### 3.4. Proof of phoresy

**Figure 10.**
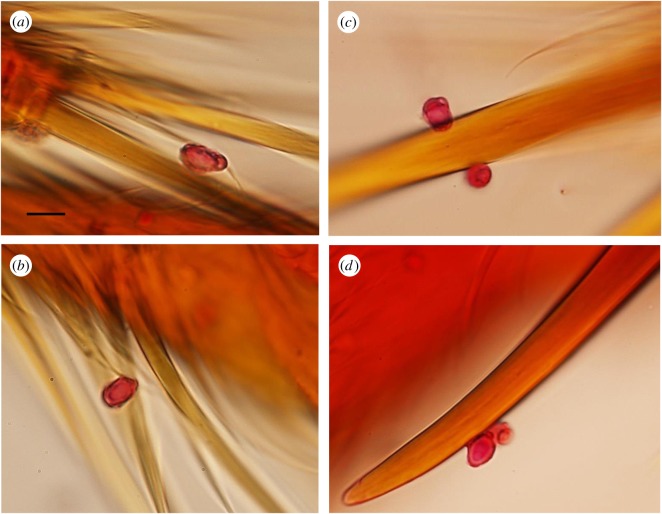
Proof of phoresy. (*a,b*) Spores of *Escovopsis moelleri* attached by apical caps to fine hairs on the lower leg (tarsus) of *Atta laevigatus*, and (*c,d*) spores attached to tarsal claws. Scale bar, (*a–d*) 10 µm.

Both laboratory and field experiments (with two Attine species, five colonies in total and two isolates of *E. moelleri*) showed that spores of *E. moelleri*, characterized by their large size (approx. 10 µm in length) and apical cap, could be found attached to both the large and small hairs on the lower leg sections (tarsi) of Attine ants (figure 10, table 1), as well as on the foot pads. Conversely, no spores were found on the control ants, except for one case from the field, in which eight *Escovopsis* spores (not *E. moellerii,* but identifiably *Escovopsis* due to the observed ornamentation) were found. Despite the large errors (table 1), there was a consistent pattern of foraging ants picking up *E. moelleri* spores across the situations, species, isolates and colonies. The ants made no attempt to avoid the discs and some were even removed and carried towards the nest by passing worker ants (figure 2*c,d*). This offers proof that the ants fail to recognize the threat posed by the dormant spores of *Escovopsis*, while the presence of *Escovopsis* spores on ‘control’ ants from the field suggests that this is indeed a natural phenomenon.

**Table 1.** Assays of phoresy of two isolates of *Escovopsis moelleri* on legs of two attine ant species in the laboratory and field (within an Atlantic Forest fragment close to Viçosa, MG, southeastern Brazil). Sand and (filter) paper refer to the substrate on to which spores were placed for ants to walk across, while *N* represents individual workers (foragers) of the given ant species and colony that were tested. *E. moelleri* codes relate to distinct isolates, each from the municipality of Viçosa—j5 is the type strain [12], while e1 was not deposited.

	colony	mean number spores (±s.e.m.)	*N*
*Acromyrmex subterraneus subterraneus* (laboratory)			
paper + *E. moelleri* (e1)	A	15.91 (± 9.31)	9
	B	13.62 (± 6.13)	8
	C	21.50 (± 10.52)	8
paper + *E. moelleri* (j5)	A	10.33 (±3.97)	9
	B	16.00 (±5.52)	8
	C	7.33 (±2.95)	9
control	A	0.00	5
	B	0.00	5
	C	0.00	5
*Atta laevigata* (field)			
paper + *E. moelleri* (e1)	a	3.00 (±1.08)	4
	b	3.66 (±2.73)	3
paper + *E. moelleri* (j5)	a	7.20 (±5.01)	5
	b	0.66 (±0.33)	3
control	a	2.00^a^ (±2.00)	4
	b	0.00	3

^a^These spores (eight in total) were identifiably *Escovopsis*, based on their ornamentation, but were not *E. moelleri*.

## 4. Discussion

Our field observations show that *Ac. subterraneus subterraneus* ants remove *Escovopsis*-infected waste from the internal fungal garden and judiciously construct external middens. The middens are built at some distance from the nest entrance and always below it. This consistency of midden location seems to be a management strategy to avoid recontamination of the nest, as posited for *At. colombica* [17]. Also, worker ants deposited the colony waste under fallen leaves whenever these were present: in effect, serving to conceal it, as predicted by Weber [18]. In a normal closed forest system, this means that the middens would be difficult to detect in the leaf litter. However, our study of nests located along a relatively steeply sided, regularly cleaned walking trail offered a unique and abnormal perspective of the activities of midden building in *Acromyrmex*. Similarly, exposed roadside sites were also selected for the study of waste management in *Ac. lobicornis* in Argentina [19], and—in the nests located on inclined ground—the middens were always constructed downslope from the nest entrance and often downwind. The authors considered this behaviour to be analogous to compartmentalization since it segregates contaminated and non-contaminated nest areas.

It has been stated that ‘The mechanism of *Escovopsis* transmission continues to be enigmatic, with untested hypotheses of commensal garden arthropods vectoring spores between colonies, or foraging ants picking up spores via encounters outside of the nest, as reasonable leads’ [11]. This is a plausible scenario that inevitably requires the mycoparasite to sporulate outside of the nest at some point. Here, we present the first evidence that *Escovopsis*—an obligate mycoparasite of the fungal garden of attine ants—can occur regularly and sporulate consistently outside the nest. There is an isolated report of a blooming event of *Escovopsis* on a large external midden of *At. colombica* in Panama, which was associated with colony emigration, but this was viewed as unusual since it had not been observed previously [20]. Significantly, the garden waste was deposited by *At. colombica* on ‘the slope of a shallow road cutting’ [20]. In our experimental site, both the nests and middens were exposed and thus prominent, quite unlike the situation in undisturbed forest systems. In the garden waste management system described for *At. colombica*—one of only two species of this genus to construct external middens—the ants, in general, drop the waste from a branch or log onto the midden [21], and there appears to be no elaborate construction, in sharp contrast with that observed here for *Acromyrmex*. It was hypothesized that this ‘elevated-dumping behaviour’ served to reduce the spread of *Escovopsis* from the midden to the fungal garden via the midden workers [17]. During another study, midden workers of *At. colombica* were intercepted on emerging from the nest and agar isolations were made from the carried material and, of the 23 ant colonies examined, 16 showed *Escovopsis* [21]. Obviously, if 70% of the middens of this ant species are being ‘inoculated’ with the mycoparasite, then the previous observation of *Escovopsis* overgrowing a midden of *At. colombica* [20] cannot be viewed as an isolated case; although such a mass sporulation (blooming) event is probably less common—as shown during our study—and dependent on specific climatic or edaphic conditions.

The ubiquity of *Escovopsis* in, and its sporulation on, middens constructed by workers of *Ac. subterraneus subterraneus* (and note that this subspecies has a wide distribution across South America and this behaviour may well be replicated in other *Acromyrmex* species) provides evidence on how the fungus can ‘escape’ from the nest and indicates a potential mechanism for transmission between nests. We do not have consistent quantitative data on prevalences within middens as we opted, in our sampling, to emphasize sampling a larger number of nests/middens rather than obtaining data from within these. Nevertheless, even though we did not submit the field-collected isolates to sequencing for full identification, we could determine that they belonged predominantly to the *apergilloides*-like morphology group (that includes *E. lentecrescens*), supported by their slow growth *in vitro*. We suspect that these slow-growing representatives of the genus employ a distinct strategy from faster-growing species, to colonize both fungal gardens and middens, a subject requiring further investigation.

Rain-splash and water run-off would appear to be the principal dispersal agents from the midden inoculum source, rather than wind, since *Escovopsis* spores are not powdery and tend to stick together. This clumping is partly the result of the distinctive wall ornamentation laid down as the spores mature. All *Escovopsis* species identified, thus far, have this characteristic—although the occurrence and significance of this particular trait has previously been overlooked in the literature, notably that relating to taxonomy [22–24]—and mature conidia are pigmented, thick-walled and conspicuously ornamented, with larger spores developing prominent and apparently sticky caps at the apices, traits that suggest both a phoretic and a survival function [12]. In fact, there is emerging evidence from ongoing SEM studies that each species of *Escovopsis* may have a distinctive pattern of ornamentation (figure 7).

We posit that the fully mature spores leached from the ephemeral middens by rain action not only contaminate the surrounding soil and leaf litter but would also be dispersed further afield by run-off during the frequent downpours in the rainy season. The dormant spores could even be channelled into neighbouring foraging trails—especially the prominent ones made by *Atta* species—and, thus, would be positioned to attach to the myriad of passing leaf-cutting ants. Notably, there is evidence that *Atta* nests can harbour both *aspergillus*- and *weberi*-like species of *Escovopsis* [8]. It has been conjectured that *Escovopsis* is ‘more likely to be transmitted between colonies by commensal arthropods’ [11], because ant foragers are unlikely to enter other nests and, moreover, can recognize such threats to the colony and remove the spores by grooming [5,6,21]. In fact, fungus-growing ants have an intricate behavioural repertoire that promotes the growth of their fungal cultivar and ensures prophylactic strategies against disease [5,25–27]. However, we argue it is more probable that the ants themselves are the primary horizontal vectors. Despite this proven ability to recognize actively metabolizing mycoparasites infesting the fungal gardens, the ability of the ants to recognize the quiescent conidia of *Escovopsis* as a specific threat is questionable, and this was demonstrated during the phoresy experiment when field-collected ants failed to remove the spores of *Escovopsis*, even when contained in tubes for extended periods prior to examination. In this experiment, the spores were found to be readily picked up by ants passing rapidly over the spore-impregnated paper discs (figure 2), and we envisage that this situation would occur naturally in the field when foraging ants move along trails contaminated with spores splashed or washed from middens. The sticky *Escovopsis* conidia may eventually become dislodged or be removed during general grooming within the nest, where they would remain dormant until chemicals from the fungal garden stimulate germination as shown by the dormancy experiment described here. We do not propose, of course, that the spore concentrations we used are realistic for a field situation; our intent was to provide proof of concept and we recognize that further work is needed on the potential route into attine colonies that we are proposing.

The dormancy experiment highlights the coevolutionary aspects of the *Escovopsis*–*Leucoagaricus* association. The spores of three different *Escovopsis* species reacted positively in the presence of the mutualistic symbiont, but not to another related agaric fungus. In fact, this consistent trait of older spores to ‘hibernate’—which, along with spore-wall ornamentation is critical to the efficiency of the transmission process—had previously been noted, albeit, inadvertently. In a study to assess the effect of faecal and mandibular gland fluids on the spore germination of micro-fungi isolated from nests of leaf-cutting ants [28], the authors concluded that the low germination rates (approx. 3.5%) of the *Escovopsis* isolates tested was due the short lifespan of their conidia. In the light of our findings, it can safely be assumed that the spores were not dead but, in fact, had entered the dormancy phase. It is also interesting to note that, when we added mycelial plugs of *Leucoagaricus* to plates where *Escovopsis* had not germinated, germination and growth ensued and were more rapid where the *Leucoagaricus* material was older. This probably reflects a greater concentration of volatiles from the older colonies, that have either had more time to accumulate or that are produced in greater quantities with age of the mycelium.

As well as *Acromyrmex*, foraging ants of *Atta* are also potential vectors of the mycoparasite into their nests and there is ample evidence of cross-infectivity of fungal gardens and thus that both genera share the same strains or morphotypes of *Escovopsis* [8,11]. More recent work has shown that *Escovopsis* strains are even shared between non-leafcutters and the leaf-cutting genera in the higher Attini [29]. All *Atta* species, apart from *At. colombica* and *At. mexicana* [30,31], have developed a sophisticated waste disposal system which ensures that any material harmful to the colony—and, of course, *Escovopsis*-infected fungal garden would fall into this category—is sealed in underground chambers [17,21], thereby eliminating the chances of contamination of both the nest and its surroundings. Nevertheless, despite this advanced waste-management strategy, the threat posed by *Escovopsis* to *Atta* colonies remains because of the less developed and generally more insanitary management of fungal garden waste by their *Acromyrmex* neighbours. In other environments, where *Acromyrmex* species may deposit waste underground, or above-ground but in arid environments that disfavour fungal growth, this may not hold. But in the humid subtropics or tropics, we posit that the waste disposal strategies of *Acromyrmex* do pose a threat to *Atta.*

To conclude, fungal parasites represent a danger to the leaf-cutting ants of the New World. Some, such as fungi of the genus *Escovopsis*, threaten the livelihoods of these insect farmers by parasitizing their mushroom symbionts (*Leucoagaricus*, Agaricales) and so are ‘weeded out’ by worker ants and ejected from the gardens; either at safe distances from the nest—as in the genus *Acromyrmex*—or sealed within it—as in most species of the genus *Atta*. However, out of sight is not out of mind since *Escovopsis* consistently sporulates on the external waste piles or middens, as shown during this study; subsequently, infesting the soil and creating potential ‘minefields’ through which neighbouring leaf-cutting ants must pass. We show that *Escovopsis* is highly evolved to maximize survival, through the thick-walled, pigmented spores and horizontal transmission, through the elaborate ornamentation of sticky caps and filaments on the spore surface. Dormancy of these phoretic spores is broken in the presence of the fungal garden symbiont, revealing a further adaptation that increases the chances of effective transmission of *Escovopsis* to its *Leucoagaricus* host. While there are many blanks to be filled in regarding the mechanism we propose for horizontal transmission of *Escovopsis*, we feel that there is much in the biology of *Escovopsis* (its presence in middens, spore ornamentation and phoresy, dormancy and its breaking) that supports the proposed mechanism. It is abundantly apparent that each species of *Escovopsis* employs a distinct strategy and this seems to be a fascinating area for future study.

## Data accessibility

Data from middens: Dryad: http://dx.doi.org/10.5061/dryad.896r3 [32].
